# Supplementary material for: End Sequence Analysis Toolkit (ESAT) expands the extractable information from single-cell RNA-seq data
Source: Genome Res. 2016 Oct;26(10):1397–410. doi: 10.1101/gr.207902.116 (PMC5052061; doi:10.1101/gr.207902.116)
Supplement: Supplemental Material [file supp_gr.207902.116_Supp_Materials.tar.gz › ESAT-esat_v0.1/doc/html/classumms_1_1core_1_1alignment_1_1_paired_read_alignment.html]

ESAT: umms.core.alignment.PairedReadAlignment Class Reference


ESAT


- Main Page
- Related Pages
- Packages
- Classes
- Files

- Class List
- Class Index
- Class Hierarchy
- Class Members

All Classes Namespaces Files Functions Variables Enumerator Pages

- umms
- core
- alignment
- PairedReadAlignment

Public Member Functions |
List of all members

umms.core.alignment.PairedReadAlignment Class Reference

Inheritance diagram for umms.core.alignment.PairedReadAlignment:

[legend]

Collaboration diagram for umms.core.alignment.PairedReadAlignment:

[legend]

|  |  |
| --- | --- |
| Public Member Functions | |
|  | PairedReadAlignment (SingleEndAlignment read1, SingleEndAlignment read2) |
|  | |
|  | PairedReadAlignment (SingleEndAlignment read1, SingleEndAlignment read2, TranscriptionRead strand) |
|  | |
| boolean | equals (Object o) |
|  | |
| int | hashCode () |
|  | |
| Public Member Functions inherited from umms.core.alignment.AbstractPairedEndAlignment | |
|  | AbstractPairedEndAlignment (BasicAnnotation b) |
|  | |
| void | refreshAttributeMap () |
|  | |
| String | getReadName () |
|  | |
| String | getChromosome () |
|  | |
| int | getMappingQuality () |
|  | |
| int[] | getIntervalBetweenReads () |
|  | |
| boolean | isPaired () |
|  | |
| boolean | isNegativeStrand () |
|  | |
| boolean | isDuplicate () |
|  | |
| void | setDuplicateFlag (boolean duplicateFlag) |
|  | |
| Alignment | getFirstMate () |
|  | |
| Alignment | getSecondMate () |
|  | |
| int | getFragmentMidpoint (Annotation annot) |
|  | |
| Collection<?extends Window > | getFragment (CoordinateSpace C) |
|  | |
| Strand | getFragmentStrand () |
|  | |
| void | setFragmentStrand (TranscriptionRead transcriptionRead) |
|  | |
| Collection< Integer > | getFragmentSize (CoordinateSpace C) |
|  | |
| int | getFragmentStart () |
|  | |
| int | getFragmentEnd () |
|  | |
| void | setAttribute (String attribute, String value) |
|  | |
| String | getAttribute (String attribute) |
|  | |
| Annotation | getReadAlignmentBlocks (CoordinateSpace C) |
|  | |
| Collection< Alignment > | getReadMates () |
|  | |
| String | toString () |
|  | |
| int | getStart () |
|  | |
| int | getEnd () |
|  | |
| String | getChr () |
|  | |
| int | getAlignmentStart () |
|  | |
| int | getAlignmentEnd () |
|  | |
| boolean | isMapped () |
|  | |
| boolean | isChimera () |
|  | |
| String | getReadSequence () |
|  | |
| double | getWeight () |
|  | |
| boolean | isProperPair () |
|  | |
| void | setProperPairFlag (boolean properPairFlag) |
|  | |
| void | shift (int delta) |
|  | |
| void | moveToCoordinate (int coord) |
|  | |
| final SAMRecord | toSAMRecord () |
|  | |
| Strand | getOrientation () |
|  | |
| Collection< Annotation > | getReadAlignments (CoordinateSpace space) |
|  | |
| boolean | hasIndel () |
|  | |
| int | getFirstFragmentPositionStranded () |
|  | |
| int | getLastFragmentPositionStranded () |
|  | |
| void | setHeader (SAMFileHeader header) |
|  | |
| SAMFileHeader | getHeader () |
|  | |
| Public Member Functions inherited from umms.core.annotation.BasicAnnotation | |
|  | BasicAnnotation () |
|  | |
|  | BasicAnnotation (SAMRecord samRecord) |
|  | |
|  | BasicAnnotation (String ucsc) |
|  | |
|  | BasicAnnotation (String referenceName, int start, int end, Strand orientation, String name) |
|  | |
|  | BasicAnnotation (String referenceName, Strand orientation, String name, Collection<?extends Annotation > blocks) |
|  | |
|  | BasicAnnotation (String referenceName, int start, int end) |
|  | |
|  | BasicAnnotation (String referenceName, int start, int end, Strand orientation) |
|  | |
|  | BasicAnnotation (String referenceName, int start, int end, String orientation) |
|  | |
|  | BasicAnnotation (BasicAnnotation other) |
|  | |
|  | BasicAnnotation (Annotation other) |
|  | |
|  | BasicAnnotation (Collection<?extends Annotation > blocks) |
|  | |
|  | BasicAnnotation (Collection<?extends Annotation > blocks, Strand orientation, String name) |
|  | |
|  | BasicAnnotation (Collection<?extends Annotation > blocks, String name) |
|  | |
|  | BasicAnnotation (String referenceName, CompoundInterval blocks, Strand orientation, String name) |
|  | |
|  | BasicAnnotation (String referenceName, CompoundInterval blocks, Strand orientation) |
|  | |
|  | BasicAnnotation (String referenceName, CompoundInterval blocks) |
|  | |
| Annotation | copy () |
|  | |
| int | getStart () |
|  | |
| int | getEnd () |
|  | |
| int | getMidpoint () |
|  | |
| String | getReferenceName () |
|  | |
| String | getName () |
|  | |
| Strand | getOrientation () |
|  | |
| boolean | isUnoriented () |
|  | |
| List<?extends Annotation > | getBlocks () |
|  | |
| List<?extends Annotation > | getBlocks (boolean oriented) |
|  | |
| Annotation[] | getFlankingBlocks (Annotation spliceJunction) |
|  | |
| int | numBlocks () |
|  | |
| int | length () |
|  | |
| int | getReferenceCoordinateAtPosition (int positionInAnnotation, boolean ignoreOrientation) |
|  | |
| int | getPositionAtReferenceCoordinate (int referenceCoordinate, boolean ignoreOrientation) |
|  | |
| double | getScore () |
|  | |
| String | toString () |
|  | |
| void | setStart (int start) |
|  | |
| void | setEnd (int end) |
|  | |
| void | setOrientation (Strand orientation) |
|  | |
| void | setOrientedStart (int orientedStart) |
|  | |
| void | setOrientedEnd (int orientedEnd) |
|  | |
| void | setReferenceName (String refName) |
|  | |
| void | setName (String n) |
|  | |
| void | setScore (double s) |
|  | |
| void | addBlocks (Annotation block) |
|  | |
| void | addBlocks (Collection<?extends Annotation > blocks) |
|  | |
| void | shift (int delta) |
|  | |
| void | moveToCoordinate (int coordinateInReference) |
|  | |
| boolean | overlaps (Annotation other, int buffer, boolean considerOrientation) |
|  | |
| boolean | overlaps (List<?extends Annotation > otherBlocks, int buffer) |
|  | |
| int | getOverlap (Annotation other) |
|  | |
| boolean | contains (Annotation other) |
|  | |
| Annotation | union (Annotation other) |
|  | |
| Annotation | intersect (Annotation other) |
|  | |
| List< Annotation > | disect (Annotation a) |
|  | |
| List< Annotation > | disect (List<?extends Annotation > disectors) |
|  | |
| Annotation | minus (Annotation other) |
|  | |
| Annotation | minus (Collection<?extends Annotation > others) |
|  | |
| void | stitchTo (Annotation next) |
|  | |
| Annotation | complement () |
|  | |
| Collection<?extends Annotation > | getSpliceConnections () |
|  | |
| Public Member Functions inherited from umms.core.annotation.AbstractAnnotation | |
| int | size () |
|  | |
| int | getSize () |
|  | |
| int | getMidpoint () |
|  | |
| Strand | getStrand () |
|  | |
| boolean | hasOrientation () |
|  | |
| boolean | isNegativeStrand () |
|  | |
| String | getChr () |
|  | |
| int | getOrientedStart () |
|  | |
| int | getOrientedEnd () |
|  | |
| int | getLengthOnReference () |
|  | |
| int | getReferenceCoordinateAtPosition (int positionInAnnotation) |
|  | |
| int | getPositionAtReferenceCoordinate (int referenceCoordinate) |
|  | |
| void | setOrientation (char orientation) |
|  | |
| void | expand (int deltaStart, int deltaEnd) |
|  | |
| Annotation | trim (int deltaStart, int deltaEnd) |
|  | |
| boolean | fullyContains (Annotation other) |
|  | |
| boolean | overlaps (Annotation other) |
|  | |
| boolean | overlaps (Annotation other, int buffer) |
|  | |
| boolean | overlapsStranded (Annotation other) |
|  | |
| boolean | overlaps (Annotation other, boolean considerOrientation) |
|  | |
| boolean | overlaps (Collection<?extends Annotation > others) |
|  | |
| boolean | overlaps (Collection<?extends Annotation > others, int buffer) |
|  | |
| String | toUCSC () |
|  | |
| String | toBED () |
|  | |
| final String | getFullInfoString () |
|  | |
| String | toBED (int r, int g, int b) |
|  | |
| String | toShortBED () |
|  | |
| String | toBEDGraph () |
|  | |
| int | getDistanceTo (Annotation other) |
|  | |
| String | toCigar () |
|  | |
| List< Annotation > | intersect (List<?extends Annotation > others) |
|  | |
| int | compareTo (Annotation other) |
|  | |
| int | compareToAnnotation (Annotation b) |
|  | |
| int | compareToAnnotation (Annotation b, boolean useOrientation) |
|  | |
| boolean | equals (Annotation a) |
|  | |
| boolean | equals (Annotation a, boolean useOrientation) |
|  | |
| int | getSAMStart () |
|  | |
| int | getSAMEnd () |
|  | |
| Public Member Functions inherited from umms.core.alignment.Alignment | |
| Collection<?extends Annotation > | getSpliceConnections () |
|  | |

|  |  |
| --- | --- |
| Additional Inherited Members | |
| Static Public Member Functions inherited from umms.core.annotation.BasicAnnotation | |
| static Annotation | createFromUCSC (String ucsc) |
|  | |
| static BasicAnnotation | fromFullInfoString (String fullInfoString) |
|  | |
| static void | main (String[] args) throws IOException |
|  | |
| Static Public Member Functions inherited from umms.core.annotation.AbstractAnnotation | |
| static Strand | getStrand (String orientation) |
|  | |
| static Strand | getStrand (char orientation) |
|  | |
| Static Public Attributes inherited from umms.core.annotation.AbstractAnnotation | |
| static final int | MAX\_DISTANCE = 1000000000 |
|  | |
| Static Protected Member Functions inherited from umms.core.alignment.AbstractPairedEndAlignment | |
| static BasicAnnotation | asAnnotation (SingleEndAlignment firstMate, SingleEndAlignment secondMate, boolean fullFragment) |
|  | |
| Protected Attributes inherited from umms.core.annotation.BasicAnnotation | |
| CompoundInterval | blocks = new CompoundInterval() |
|  | |

## Detailed Description

Author
:   prussell Alignment of two paired reads without the fragment in between

## Constructor & Destructor Documentation

|  |  |  |  |
| --- | --- | --- | --- |
| umms.core.alignment.PairedReadAlignment.PairedReadAlignment | ( | SingleEndAlignment | *read1*, |
|  |  | SingleEndAlignment | *read2* |
|  | ) |  |  |

Constructs a paired end alignment object from two alignments.

Parameters
:   |  |  |
    | --- | --- |
    | read1 |  |
    | read2 |  |

Here is the caller graph for this function:

|  |  |  |  |
| --- | --- | --- | --- |
| umms.core.alignment.PairedReadAlignment.PairedReadAlignment | ( | SingleEndAlignment | *read1*, |
|  |  | SingleEndAlignment | *read2*, |
|  |  | TranscriptionRead | *strand* |
|  | ) |  |  |

Constructs a paired end alignment object from two alignments and provides the read that is in the direction of transcription

Parameters
:   |  |  |
    | --- | --- |
    | read1 |  |
    | read2 |  |
    | strand |  |

Here is the call graph for this function:

## Member Function Documentation

|  |  |  |  |  |  |
| --- | --- | --- | --- | --- | --- |
| boolean umms.core.alignment.PairedReadAlignment.equals | ( | Object | *o* | ) |  |

Here is the call graph for this function:

|  |  |  |  |  |
| --- | --- | --- | --- | --- |
| int umms.core.alignment.PairedReadAlignment.hashCode | ( |  | ) |  |

Here is the call graph for this function:

---

The documentation for this class was generated from the following file:

- java/umms/core/alignment/PairedReadAlignment.java


---

Generated on Fri Aug 15 2014 15:11:07 for ESAT by  

 1.8.7
